# Fungal Endophytes: A Promising Frontier for Discovery of Novel Bioactive Compounds

**DOI:** 10.3390/jof7100786

**Published:** 2021-09-22

**Authors:** Martin Muthee Gakuubi, Madhaiyan Munusamy, Zhao-Xun Liang, Siew Bee Ng

**Affiliations:** 1 Singapore Institute of Food and Biotechnology Innovation (SIFBI), Agency for Science, Technology and Research (A*STAR), 31 Biopolis Way, #01-02 Nanos, Singapore 138669, Singapore; tp-gakuubimm@sifbi.a-star.edu.sg (M.M.G.); madhaiyanm@sifbi.a-star.edu.sg (M.M.); 2 School of Biological Sciences, Nanyang Technological University, Singapore 637551, Singapore; zxliang@ntu.edu.sg

**Keywords:** bioactive compounds, endophytic fungi, natural products, secondary metabolites

## Abstract

For years, fungi have served as repositories of bioactive secondary metabolites that form the backbone of many existing drugs. With the global rise in infections associated with antimicrobial resistance, in addition to the growing burden of non-communicable disease, such as cancer, diabetes and cardiovascular ailments, the demand for new drugs that can provide an improved therapeutic outcome has become the utmost priority. The exploration of microbes from understudied and specialized niches is one of the promising ways of discovering promising lead molecules for drug discovery. In recent years, a special class of plant-associated fungi, namely, fungal endophytes, have emerged as an important source of bioactive compounds with unique chemistry and interesting biological activities. The present review focuses on endophytic fungi and their classification, rationale for selection and prioritization of host plants for fungal isolation and examples of strategies that have been adopted to induce the activation of cryptic biosynthetic gene clusters to enhance the biosynthetic potential of fungal endophytes.

## 1. Introduction

Natural products, also known as secondary metabolites (SMs), are a diverse group of mainly low-molecular-weight and structurally diverse chemical entities that are produced by almost all living organisms, but mostly by microbes [1]. The use of natural products as a source of medicine dates back several millennia and is extensively described in the literature of ancient civilizations. There is evidence that the Chinese, Indians and Egyptians were using plants and plant extracts for treatment of human diseases thousands of years ago [2]. While plants have a long history as a source of beneficial SMs, microorganisms have equally played a key role in the evolution of drug discovery due to their impressive diversity, ease of growth and amenability to genetic manipulation. 

In the early days of microbial-based drug discovery, the soil microbiota was the principal source of bioactive compounds [3,4]. However, with the increase in the rate of rediscovery of known compounds from soil microbes, there has been a need to widen the horizon in search of new sources of microbes with greater potential for discovery of novel therapeutic candidates. Among the most promising sources of microbes are those from underexplored and specialized niches, including plant-associated microbes, such as endophytes. Already, fascinating compounds with great therapeutic value such as anticancer agents, antibiotics, antiviral, antidiabetic and immunosuppressive compounds have been isolated from these largely untapped microbes [5,6,7,8].

## 2. Fungal Endophytes

Endophytes refers to a group of highly diverse microorganisms, often fungi and bacteria, that are found in association with plants. The term endophytes (*endo* = inside; *phytón* = plant) was coined by Anton de Bary in 1866 to denote fungi that live inside their host plant tissues—as opposed to ‘epiphytes’, a term assigned to fungi that are found on the surface of plants [9]. Endophytic fungi constitute a heterogeneous and diverse group of microbes that are found in essentially all types of plants growing in different climatic and ecological zones [10]. Fungal endophytes have been isolated from virtually all plant types including trees, shrubs, herbaceous plants, grasses, mosses and ferns and marine plants [11]. The number of endophytic fungi is estimated to be approximately one million based on the 6:1 ratio of fungal–plant species [12]. However, the actual figure may be much higher than this, given the fact that only a small fraction of the approximately 5% of fungal species have been described to date [13]. Furthermore, the validity of the 6:1 ratio has been questioned, with some authors suggesting that the ratio could be up to five times higher in areas such as tropical and subtropical regions [14].

Fungal endophytes were originally defined as fungal strains that colonize internal plant tissues without causing apparent harm or disease to their host [12,15]. However, this definition has evolved over the years and will undoubtedly undergo more redefinitions as more data continue to be generated from research in endophyte biology. First, it can be quite difficult at times to make a clear distinction between fungal endophytes, mycorrhizal and phytopathogenic fungi. For example, some fungal strains can be pathogenic to certain plants while having neutral or even beneficial qualities towards other host plants [16]. Some fungi have also been known to live as latent pathogens within their host plant tissues only for their pathogenicity to be activated once certain conditions become favorable [17]. Additionally, there is a grey area with regard to plant pathogens that have lost their virulence. Should they be considered as endophytes or pathogens? Moreover, the definition of fungal endophytes on the basis of pathogenicity rules out unculturable fungi whose phytopathogenicity may be difficult to ascertain [16]. 

Endophytic fungi consist primarily of members of *Ascomycetes* and occasionally *Basidiomycetes* and *Zygomycetes* [12]. The diversity of fungal endophytes varies greatly by location and is a function of the environment with all its variables, host plants’ genotype and physiology, growth season, and anthropogenic influences, among other factors [18,19]. Even within the same geographical region, the diversity and abundance of endophytic fungi varies considerably among host plants, and even within different parts of the same host plants [20]. It has, for instance, been established that the diversity of fungal endophytes is much greater in the tropical and subtropical regions compared to other regions [18,21] Additionally, seasonal variations have also been cited as a major determinant of the distribution and diversity of fungal endophytes [19,22]. 

## 3. Classification of Fungal Endophytes

Being a taxonomically diverse and polyphyletic group, endophytic fungi classification can be quite challenging. Numerous schemes have been adopted in classifying fungal endophytes on the basis of one or more aspects of their biology [23]. The earliest classification scheme for fungal endophytes is based on the phylogeny, ecological functions and life history strategies and categorized fungal endophytes into two main groups: the clavicipitaceous endophytes (C-endophytes) and nonclavicipitaceous endophytes (NC-endophytes) [23,24,25]. Clavicipitaceous endophytes, also referred to as the endophytes of grasses or class 1 endophytes, consist mainly of member belonging to the family *Clavicipitaceae* (*Hypocreales*; *Ascomycota*). This group consists of 27 known genera that include the free living and diverse collection of symbiotic species [24]. Nonclavicipitaceous endophytes on the other hand consist of a vastly diverse polyphyletic group whose members are not well defined taxonomically [25]. NC-endophytes are found in association with vascular and non-vascular plants, with the majority of its members belonging to the *Dikarya* subkingdom (*Ascomycota* or *Basidiomycota*). 

With the continued growth in the field of endophyte fungal biology, a number of other criteria for classifying endophytic fungi have emerged. These include classification on the basis of characteristics such as host range, mode of transmission and infection, reproduction strategies, host’s sites of colonization, and the position of the fungi within the continuum of interactions with their hosts [23,24]. Fungal endophytes have, for instance, been classified on the basis of the host plant types and, thus, there are studies that have focused on fungal endophytes from specific plants, e.g., medicinal plants fungal endophytes [26], mangrove fungal endophytes [27], coniferous trees fungal endophytes [19] and fungal endophytes of grasses [28]. With regard to the modes of transmission, two main categories of fungal endophytes are recognized, namely, vertically and horizontally transmitted fungal endophytes. The former consists of fungal strains that are transmitted between host generations, mostly through seeds, while the latter consists of fungi that are transmitted between different individuals of a population through spores or other vegetative propagules [29].

Fungal endophytes have similarly been classified on the basis of their preferential colonization of specific host plant tissues. Studies have shown that most often, fungal endophytes exhibit preferential tissues colonization, whereby specific fungal strains are most likely to be localized in specific parts of the host plant rather than colonize the host systemically [23]. Thus, there are root endophytic fungi which are mostly isolated from host plants’ roots [30] and foliar fungal endophytes which are found in hosts’ stems and leaves [31]. Other common criteria for classifying fungal endophytes include their mode of reproduction, whereby fungal endophytes are classified as either sexual or asexual and with regard to the expression of infection, hence the differentiation between symptomatic and asymptomatic fungal endophytes [32]. 

## 4. Isolation of Fungal Endophytes: Rationale for Plant Selection and Prioritization 

Practically, all of the higher plant species in natural ecosystems harbor one to several hundred species of endophytic fungi [20]. With such a high number of possible sources of fungal endophytes, ingenious strategies have to be adopted when prioritizing the selection of host plants for fungal isolation. This increases the likelihood of selecting host plants that might harbor uncharacterized fungi, thus, enhancing the prospect of discovering interesting molecules. Though no formalized guidelines exist on the selection of plant sources for the isolation of fungal endophytes, various authors have proposed some bioprospecting strategies that coupled with statistical and comparative analysis of the ever-increasing body of literature can be used to identify the most promising host plants.

A four-point set of criteria has been proposed that can be used as a rationale for selection of promising plant for the isolation of fungal endophytes [33]. It involves the selection of (i) plants from a distinctive ecological niche, unique biology and survival strategies; (ii) medicinal plants and plants of ethnobotanical importance; (iii) plants that are endemic to specific regions; and (iv) plants from biodiversity hotspots. The first consideration gives prominence to plants from unique biotopes and those with inimitable biology and survival strategies. The idea here is that such plants may have evolved some distinctive survival strategies, allowing them to host an equally rare collection of microbial endophytes. An example of such plants would be those that grow in the mangrove environment. Such plants have evolved unique morphological, ecological and physiological adaptations to extreme conditions of high salinity, fluctuating sea water levels, high temperature and constant water forces [34]. Indeed, mangrove plants have been found to host a collection of fungal endophytes that are capable of synthesizing a rich repertoire of bioactive secondary metabolites [27,35].

Focusing on medicinal plants is another important proposition [20,36]. Of particular interest are plants with known ethnopharmacological usage among various local communities. The selection of such plants may be carried out directly through contact with the local communities, through ethnobotanical surveys studies or via a literature search. The aim is to assess whether the activity attributed to such plants may have anything to do with their associated fungal endophytes [37]. The focus on medicinal plants as a source of fungal endophytes in bioprospecting for bioactives has gained traction in recent years following the discovery of fungal strains that are capable of independently producing host-associated bioactive compounds [38,39]. Among the most well-known cases is that of an anticancer agent paclitaxel (trade name Taxol). Discovered originally from the bark of *Taxus brevifolia*, taxol remains one of the most potent anti-cancer agents. However, the quantity of the active ingredient in *T. brevifolia* is very low, making this source of the compound unsustainable [40]. A breakthrough came as a result of the discovery of paclitaxel-producing endophytic fungi *Taxomyces andreanae* from *T. brevifolia*, followed by the discovery of more paclitaxel-producing fungal endophytes from other *Taxus* and non-*Taxus* species [41,42]. Since then, other fungal endophytes have been characterized that are capable of producing similar bioactive molecules as their host plants (Table 1).

The isolation of fungi from endemic plants is another strategy that can enhance the prospect of discovering interesting fungal strains [33]. Having occupied a distinctive geographical area for a long time, many endemic plants may be host to a unique assortment of fungal endophytes with capacity for the biosynthesis of molecules with fascinating chemistry and activities [68]. The last approach is the prioritization of plants from biodiversity hotspots. Global biodiversity hotspots, such as tropical and subtropical regions, are host to the highest plant diversity [20]. Plants growing in such areas are subjected to a multitude of biotic and abiotic factors, competition for limited resources and high levels of selection pressure [69]. Over the years, such an environment is likely to have resulted in the emergence of unique and uncharacterized endophytic fungi.

## 5. Bioactive Secondary Metabolites from Endophytic Fungi 

**Figure 1:**
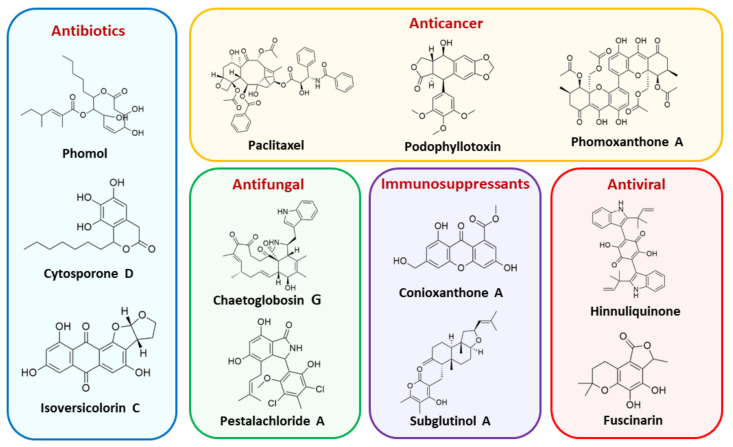
Some bioactive compounds isolated from fungal endophytes grouped according to their bioactivities.

The capacity for biosynthesis of bioactives both beneficial (such as antibiotics, e.g., penicillin) and detrimental (such as mycotoxins, e.g., aflatoxin) by fungi has motivated a great deal of interest among natural product researchers. Endophytic fungi represent an inexhaustible source of important metabolites with a broad range of biological activities. The non-pathogenic nature of the majority of fungal endophytes makes most of the SMs derived from them more suited for human usage, as most are non-toxic to mammalian cells [70]. Some of the biologically active compounds that have been isolated from fungal endophytes include antibiotics, anticancer, antifungal, immunosuppressive and antiviral compounds (Figure 1). The extensive review articles have been published focusing on major classes of bioactive compounds sourced from fungal endophytes including antibiotics [71,72], antifungal compounds [73], antimycobacterial compounds [74], anticancer/cytotoxic compounds [25,75,76], antioxidants [77] and valuable enzymes [78,79]. 

## 6. Enhancement of Secondary Metabolite Biosynthesis in Fungal Endophytes

**Figure 2:**
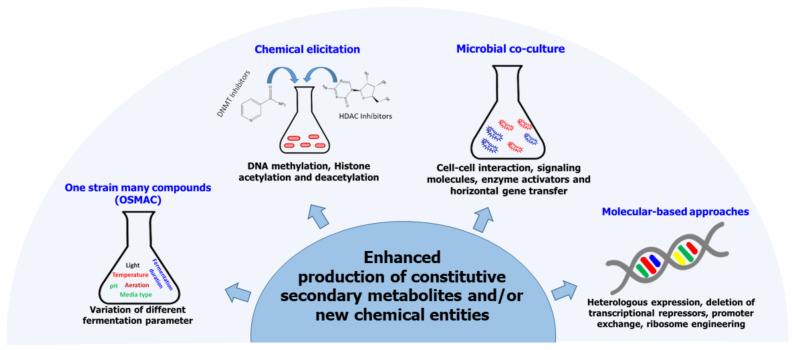
Some approaches that have been employed to enhance secondary metabolites biosynthesis in fungal endophytes.

For a long time, the consensus was that fungal genes responsible for secondary metabolism are scattered throughout the organisms’ genome in a similar manner to the genes involved in primary metabolism [80]. With the advancement in genomics, this notion of the architecture of fungal SMs biosynthetic pathways has since been dispelled, and two main principles have since been established: (i) genes that encode the enzymes needed for the biosynthesis and transport of SMs as well as pathway-specific regulatory genes are clustered on a single gene locus, forming biosynthetic gene clusters (BGCs) [81,82]. This design of fungal SMs biosynthetic genes has enabled the development of computer algorithms that can predict the core enzymes involved in the biosynthesis of various classes of SMs [83]. (ii) The number of biosynthetic gene clusters outnumbers that of SMs detected for any fungal strain by far [80,84]. The inconsistency between the actual number of BGCs and the number of characterized chemical entities produced by any given fungi is attributed to the fact that most BGCs remain silent or are only weakly expressed under standard laboratory growth conditions [85,86]. Arising from this observation, therefore, is the need to come up with techniques that can be used to induce the activation of cryptic biosynthetic pathways to enhance SMs biosynthesis in fungal endophytes. Below, we highlight some of the approaches that have been employed to activate silent gene cluster in fungal endophytes (Figure 2).

### 6.1. One-Strain Many-Compound (OSMAC) Approach

The one-strain many-compound (OSMAC) concept was coined following an observation that changes in various cultivation parameters are capable of altering the SM biosynthetic profiles of microbes, leading to the discovery of new compounds [87]. Through systematic manipulation of media composition, aeration, culture vessel and use of selected enzyme inhibitors, up to 20 different SMs were isolated from a single microbe [87]. Similar results have been obtained in other studies through manipulation of the abovementioned among other culture growth conditions. Such changes have been found to induce changes not only in the amount of a specific metabolites, but have, in many instances, resulted in the biosynthesis of novel chemical entities altogether [88,89].

While manipulation of media composition has traditionally been used as a tool to optimize the production of already known metabolites, this technique has recently found widespread application as a mean of awakening cryptic or poorly expressed BGCs in fungal endophytes. In one such study, growth of *Pestalotiopsis photiniae*, an endophytic fungus isolated from *Roystonea regia* in SA media resulted in the isolation of Photinides A-F [90]. The growth of the same strain in solid rice media under static conditions resulted in the production of two new δ-lactone derivatives: photipyrones A and B together with four known analogues of the two compounds that were not produced by the strain grown in SA media [91]. In another study, *Bulgaria inquinans*, an endophytic fungus isolated from *Viscum album*, was fermented in solid Czapek medium and later in the same media supplemented with a mixture of MgSO_4_, NaNO_3_ and NaCl salts [92]. Numerous compounds were identified from the cultures grown in salt-supplemented Czapek media that were not detectable in cultures grown in normal Czapek media. Among the isolated new compound was bulgarilines B, which showed strong cytotoxic activity against the murine lymphoma cell line, L5178Y. In another OSMAC-driven study, production of metabolites by endophytic fungi *Dothideomycetes* sp. CRI7 isolated from the roots of *Tiliacora triandra*, were found to vary considerably not only on the basis of the culture media but also the source of the malt and potato ingredients in Czapek malt agar and PDB media, respectively [93]. When fermented in PDB medium prepared from fresh potato tubers, *Dothideomycetes* sp. CRI7 produced a tricyclic polyketide and three azaphilone derivatives. The same strain when grown in PDB media prepared from commercial potato powder produced three hitherto-unknown compounds that were absent from cultures grown in PDB prepared from fresh potato tubers. Furthermore, the growth of the fungal strain in Czapek malt media prepared from commercial malt extracts sourced from different countries completely altered *Dothideomycetes* sp. CRI7’s metabolite profile. Some of the isolated compound exhibited cytotoxic activity against three cancer cell lines, while one had the radical scavenging and aromatase inhibitory activity [93]. The OSMAC growth strategy was found to alter the SM biosynthetic profile of *Clonostachys rosea* B5-2, an endophytic fungus isolated from mangrove plants. The addition of apple juice into the solid rice medium induced the production of a known compound, (-)-vertinolide in addition to four novel compounds, namely, (-)-dihydrovertinolide and clonostach acids A, B and C. (-)-dihydrovertinolide exhibited moderate phytotoxic activity against lettuce seedlings [94].

Even the type of water used for media preparation can result in a remarkable change in the biosynthetic profile of fungal endophytes. Endophytic fungus *Paraphaeosphaeria quadriseptata* produced different types and quantities of secondary metabolites when grown in PDB medium prepared with tap and when the same media was prepared using distilled water [95]. These changes were attributed to the presence of certain traces ions such as Cu^2+,^ Cd^2+^ and Cr^3+^ in tap water. Another factor that has been shown to influence metabolite production among fungal endophytes is the volume of the growth culture. In one study, fermentation of fungal strains in microtiter plates (1mL of media per well) led to the biosynthesis of numerous antifungal metabolites that were not detected when the same strains were grown in 1-liter culture flasks [96]. Variations in the incubation temperature, type and intensity of light have likewise been found to induce or suppress the biosynthesis of specific SMs in fungal endophytes. For example, whereas the production of mycotoxin sterigmatocystin in *Aspergillus* sp. is repressed by white light [97], the same type of light is known to induce the biosynthesis of two mycotoxins, namely, alternariol and altertoxin in *Alternaria alternata* [98]. Other cultivation parameters that have been found to influence fungal SMs biosynthesis include the incubation time, media pH, oxygen concentration and level of aeration and growth of fungal strains in either shake flasks or static cultures [96,99,100].

**Figure 3:**
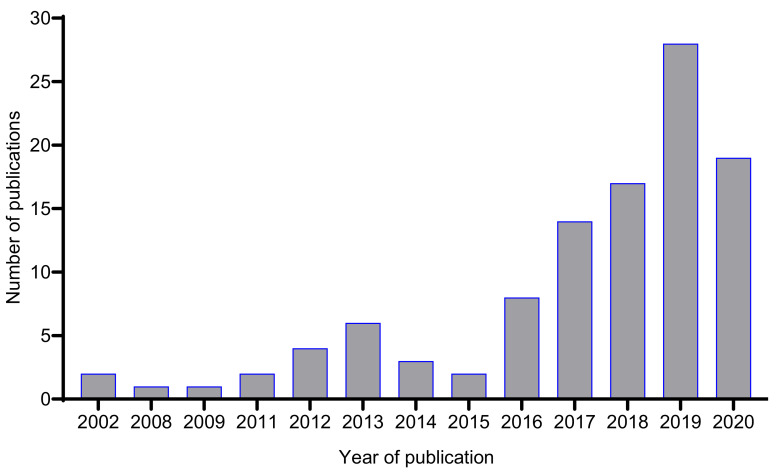
Number of journal articles published between 2002–2020 containing the phrase “one strain many compounds”, “one strain-many compounds” or “OSMAC” within the title, abstract or as a keyword (data based on a search from the Web of Science, 14 January 2021).

Since its conceptualization, OSMAC has become a mainstream approach in microbial natural product research. Figure 3 shows that, within the first five years of the introduction of the term OSMAC (2002–2006), only two journal articles containing the phrase “one strain many compounds” or “OSMAC” within the title, abstract or as keywords were published, compared to 86 papers that have been published in the last five years (2016–2020).

### 6.2. Microbial Co-Culture Approach

In their natural habitats, microorganisms exist in complex multispecies communities consisting of members of their own and those of other populations. Such communities are characterized by intricate intra- and inter-species interactions that are in stark contrast with the mainly axenic laboratory cultivation systems [88,101]. These natural microbial communities consist of complex and dynamic interactions that can fall anywhere within the continuum of mutualism and antagonism, with most having arose as strategies for defense and/or competition for limited resources among other factors [102]. In a number of instances, it has been proven that the activation of cryptic BGCs among certain microbes require an ecological context such as closer interaction with other microbes, host plants for the plant-associated microorganisms and even animals [101,103,104]. Co-culture, also referred to as co-cultivation (for solid media) or mixed fermentation (for liquid media), is the growth of two or more microbial strains together with the aim of mimicking the myriad of interactions that occurs when microbes coexist naturally [102,105]. This social networking among microbes in nature is facilitated among other things by gene transfers and production of small diffusible signaling molecules that individual strains release into their surroundings [106]. Furthermore, by taking up specific resources and releasing various metabolites and chemicals, microorganisms modify their surroundings, which affect their growth and that of other resident microbes. Such close associations between microorganisms have been shown to lead to the alteration of the biosynthetic profile of the involved strains so often, sometimes resulting in the production of compounds that are not produced in monocultures [1,96,101]. This aspect has been exploited in natural product research through co-culture of selected microorganisms, either to enhance the biosynthesis of already identified metabolites that are produced in small quantities in axenic cultures or for induction of silent gene clusters resulting in the production of novel chemical entities altogether.

**Figure 4:**
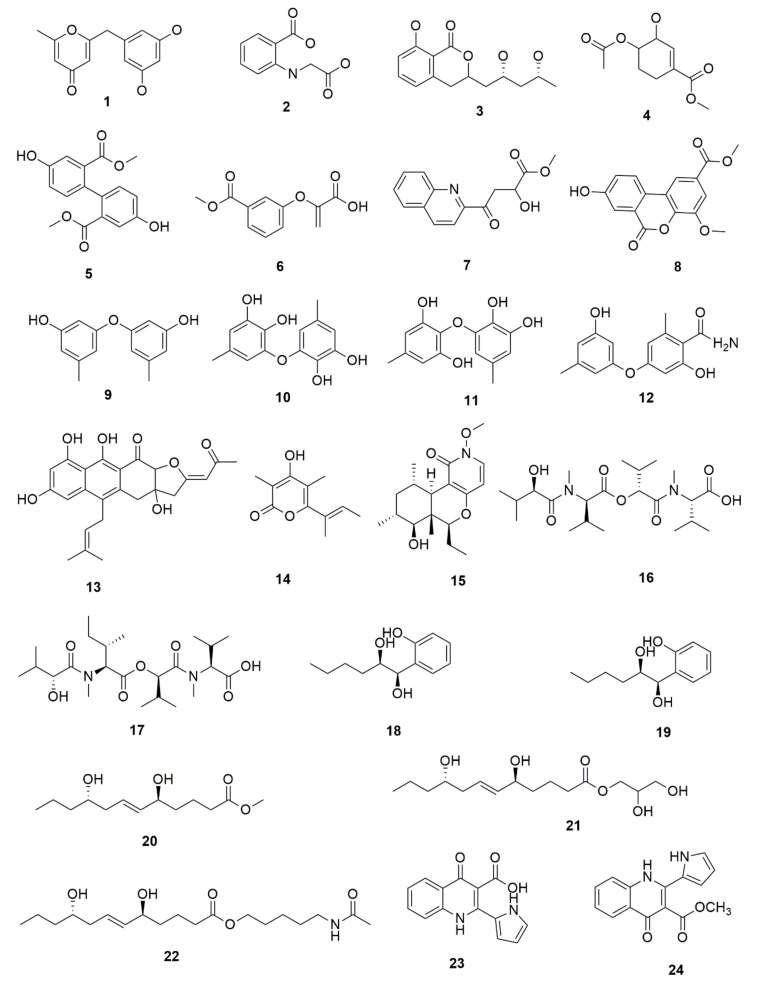
Some of the new compounds that have been isolated as a result of co-cultures of endophytic fungi with bacteria or with other fungal strains.

Fungus–bacterium and fungus–fungus are the two main co-culture schemes that have been employed for the induction of cryptic BGCs in fungal endophytes. A co-culture of endophytic fungus *Fusarium tricinctum* with a bacterium *Bacillus subtilis* led to a 78-fold increase in constitutive fungal secondary metabolites in addition to production of four compounds which were not detected in either of the monocultures, three of these compounds—macrocarpon C (**1**), 2-(carboxymethylamino) benzoic acid (**2**) and (-)-citreoisocoumarinol (**3**) of which were novel natural products [107] (Figure 4). Some of the isolated compounds, such as Enniatins B1, A1 and lateropyrone, exhibited good antibacterial activities with the first two inhibiting the growth of the inducing *B. subtilis* bacterial strain [107]. In another study, a co-culture of *Chaetomium* sp., an endophytic fungus isolated from *Sapium ellipticum*, with *B. subtilis*, resulted in an 8-fold upsurge in biosynthesis of different SMs. Seven compounds, five of which were novel, namely, shikimeran A (**4**), bipherin A (**5**), chorismeron (**6**), quinomeran (**7**) and serkydayn (**8**), were recovered from the co-cultures but not in axenic cultures [108]. In another fungal–bacterial co-culture experiment, *Aspergillus austroafricanus*, a fungal endophyte isolated from *Eichhornia crassipes*, was grown in the presence of either *B. subtilis* or *Streptomyces lividans*. While the fungal monoculture resulted in the production of two new xanthone dimers—austradixanthone and sesquiterpene (+)-austrosene, among other known compounds—a co-cultivation of the fungus with *B. subtilis* or *S. lividans* resulted in the biosynthesis of many diphenyl ethers, such as diorcinol (**9**), violaceol I (**10**) and violaceol II (**11**), in addition to a new austramide (**12**) [109]. Compound (**9**) exhibited antibacterial activity against a *S. subtilis* strain that had been used in the co-culture, while compounds (**10**) and (**11**) exhibited growth-inhibitory activities against *Staphylococcus aureus* [109]. A bacterium, *Streptomyces rapamycinicus*, when co-cultured with the fungus *Aspergillus fumigatus*, resulted in the activation of a silent PKS in the fungus, resulting in the production of a novel compound, fumicycline A (**13**) [110]. In another co-culture study involving the same two microorganisms, *S. rapamycinicus* activated a silent gene cluster in the fungus, resulting in induced biosynthesis of a novel metabolite, fumigermin (**14**), by the fungus [101]. Interestingly, the new compound, which is structurally similar to bacterial germicidins, was found to reversibly inhibit the germination of the spores in the inducing bacteria [101]. Furthermore, in both studies, the induced biosynthesis of the new metabolites required an intimate physical contact between the co-cultured microorganisms, ruling out the involvement of diffusible signaling molecules from the inducing bacteria [101,110].

Fungal–fungal co-cultures have, likewise, been used to enhance SM production in fungal endophytes. *Paraconiothyrium* sp., a paclitaxel-producing endophytic fungus, when co-cultured with *Alternaria* sp., another endophytic fungus, led to a 3-fold increase in the production of paclitaxel [111]. Furthermore, when *Phomopsis* sp., another endophytic fungus was included in the co-culture, there was an up to 7.8-fold upsurge in the production of paclitaxel recorded in comparison to the amount obtained when *Paraconiothyrium* sp. was grown axenically [111]. A co-culture of two endophytic fungi—*Epicoccum sorghinum* FT1062 and *Camporesia sambuci* FT1061, isolated from *Rhodomyrtus tomentosa—*induced the production of a novel *N*-methoxypyridone analog (**15)**] in addition to four other known compounds that were absent in the two monocultures [112]. In another study, a co-culture of two endophytic fungi *Fusarium tricinctum* and *Fusarium begonia* led to the biosynthesis of two novel depsipeptides, namely, subenniatin A (**16**) and subenniatin B (**17**), none of which were detected in axenic cultures [113]. The two compounds, however, showed no antibacterial or cytotoxic activity against a panel of bacterial pathogens and cancer cell lines, respectively [113]. A mixed fermentation of *Phoma* sp., a fungal endophyte and *Armillaria* sp., fungal symbiont resulted in the biosynthesis of five new compounds, including two phenolic compounds, namely, phexandiol A (**18**) and B (**19**), and three aliphatic ester derivatives, designated phomester A (**20**), B (**21**) and C (**22**). None of these compounds, however, exhibited any significant antimicrobial or cytotoxic activity [114]. In another study, the growth of two marine-derived endophytic fungi isolated from South China Sea (strain numbers 1924 and 3893) together yielded a novel 1-isoquinolone, marinamide (**23**) and its methyl ester (**24**). Both compounds revealed comparable antibacterial activity and potent cytotoxic activity against selected cancer cell lines [115,116].

Co-cultivation is increasingly emerging as a powerful tool for unlocking the chemical diversity of fungal endophytes by enhancing the expression of cryptic or weakly expressed biosynthetic pathways. Co-culture has proven to be a particularly popular approach for enhancing the chemical diversity of microbial SMs, owing to its simplicity and low cost when compared with other more complex gene manipulation techniques. However, when working with many target strains, the method requires large screening of diverse strain combinations before coming up with the most promising combinations, a process that can be quite cumbersome. 

### 6.3. Chemical Epigenetic Modification 

Like all other living organisms, fungi are ingrained with an intricate network of multi-level regulatory systems that govern gene expression. These control mechanisms, though essential for normal growth and development, place some restrictions on secondary metabolism. The regulation of fungal secondary metabolism is a complex process that is dependent upon an intricate network of cellular, chemical and genetic determinants [117]. It occurs via the cluster-specific regulators, as well as globally acting regulators. The latter are usually encoded by genes that are not associated with any specific gene cluster and are mediated by a wide range of signals or triggers [118,119]. Chromatin-level control of gene silencing or activation has been identified as one of the mechanisms involved in the regulation of fungal SMs biosynthesis [83,118]. One approach that has been shown to allow fungi to circumvent such regulatory roadblocks is through the use of small chemical molecules known as epigenetic modifiers [1,108]. Remodeling of the chromatin landscape by chemically targeting the histone and DNA post-translation modifications has been found to enhance the quantity of constitutive fungal secondary metabolites through the activation or suppression of SMs encoding gene clusters [120].

**Figure 5:**
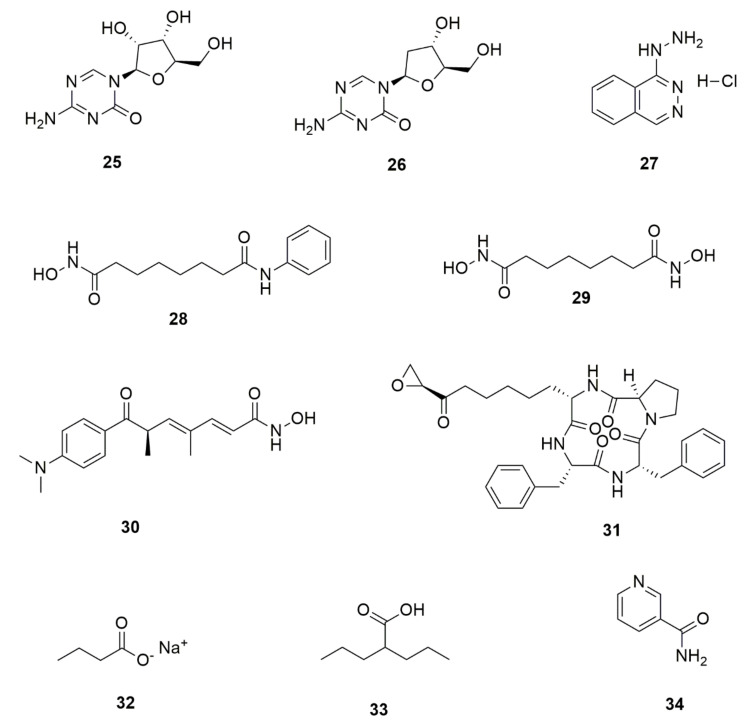
Some chemical epigenetic modifiers that have been used in fungal natural products research: DNA. methyltransferase inhibitors (**25**–**27**) and histone deacetylase inhibitors (**28**–**34**).

Chemical epigenetic modification is achieved through the cultivation of target strains in the presence of one or more of the chemical epigenetic modifier compounds, such as histone deacetylases (HDAC) inhibitors and DNA methyltransferases (DNMT) inhibitors. The addition of such compounds in growth cultures at micromolar or even nanomolar concentrations has been found to suppress or activate the associated enzymes, resulting in the reengineering of SMs biosynthetic pathways in fungi [121,122]. Figure 5 shows some of the chemical epigenetic modifiers that have been used in remodeling fungal secondary metabolism: 5-azacytidine (**25**), 5-aza-2′-deoxycytidine (decitabine) (**26**), and hydralazine hydrochloride (**27**), which are DNMT inhibitors. HDAC inhibitors, on the other hand, include suberoylanilide hydroxamic acid (vorinostat or SAHA) (**28**), Suberoyl bishydroxamic acid (SBHA) (**29**) trichostatin A (**30**), trapoxin B (**31**) sodium butyrate (**32**) valproic acid (**33**) and nicotinamide (**34**) [100,123].

**Figure 6:**
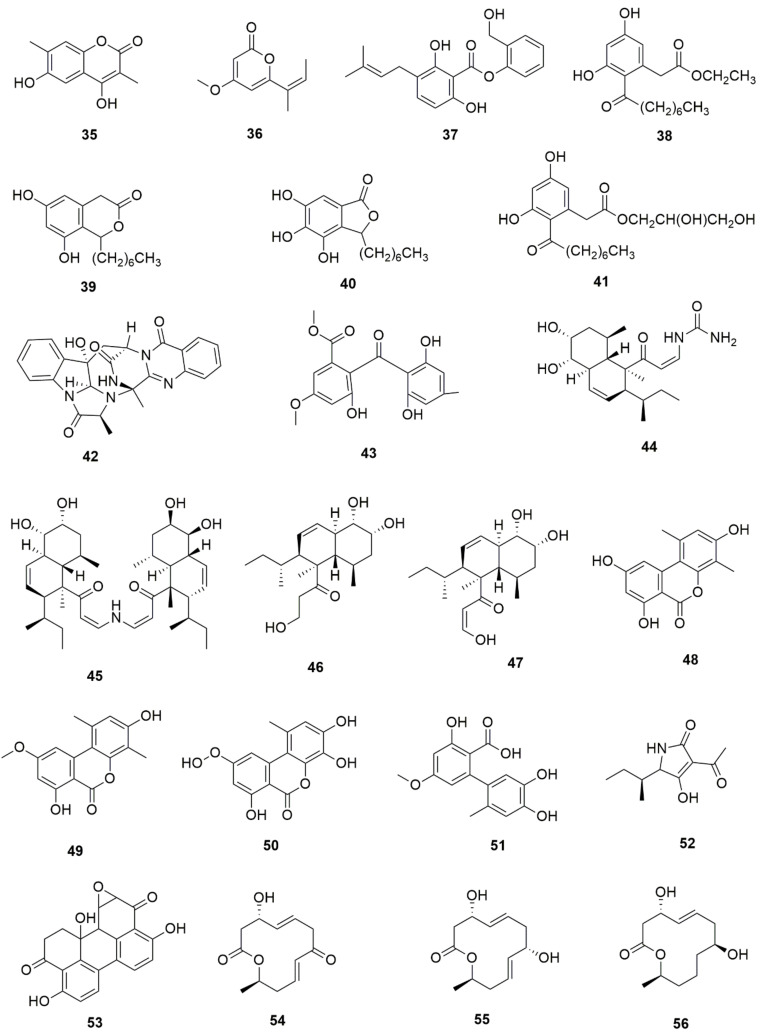
New compounds and those whose quantities have been reported to be enhanced by the growth of fungal endophytes in the presence of chemical epigenetic modifiers.

*Pestalotiopsis crassiuscula*, an endophytic fungus isolated from *Fragaria chiloensis*, when grown in PDB in the presence of 500 µM 5-azacytidine, resulted in drastic chemical differences in the resultant extracts in comparison with those from the fungi grown in the absence of the DNMT inhibitor [121]. Three novel compounds, (**35**), (**36**) and (**37**), were produced as a result of chemical elicitation (Figure 6). However, none of the three compounds showed antifungal activity against selected fungal pathogens. In another study, two epigenetic modifiers, 5-azacytidine and sodium butyrate, were added separately and in combination at varied concentrations in cultures of *Leucostoma persoonii*, a mangrove-derived fungal endophyte [124]. Overall, HDAC-inhibited cultures enhanced the levels of three previously known compounds, cytosporone B (**38**), cytosporone C (**39**) and cytosporone E (**40**), and also resulted in the isolation of a previously uncharacterized cytosporone R (**41**). Cytosporone E displayed inhibitory activity against *Plasmodium falciparum* in addition to antibacterial activity and inhibition of biofilm formation in MRSA [124]. The growth of endophytic fungus *Aspergillus fumigatus,* isolated from *Grewia asiatica* in PDB containing 500 μM of valproic acid, resulted in a decrease in the quantity of numerous compounds that were identified from the control culture. However, the concentration of fumiquinazoline C (**42**) was found to increase ten-fold in cultures of strain grown in the presence of the HDAC inhibitor [122]. A study exploring the effect of epigenetic modification and co-cultivation of *Chaetomium* sp., an endophytic fungus with *B. subtilis*, revealed the production of isosulochrin (**43**) when the fungus was grown in solid rice media in the presence of either SAHA or 5-azacytidine. Furthermore, while compound (**43**) was detected in the co-culture of *Chaetomium* sp., with the bacterium, the two chemical elicitors were found to greatly enhance its accumulation [108]. The treatment of *Eupenicillium* sp., an endophytic fungus derived from *Xanthium sibiricum* with nicotinamide, an NAD+-dependent histone deacetylase inhibitor, resulted in the production of two novel compounds designated as eupenicinicol C (**44**) and D (**45**), as well as two known and biosynthetically related compounds, eujavanicol A (**46**) and eupenicinicol A (**47**). Compound (**45**) showed inhibitory activity against *S. aureus* and some noticeable cytotoxic activity against the human acute monocytic leukemia cell line (THP-1) [125].

Manipulation of the epigenetic environment using chemical elicitors has also been reported to induce the production of toxic SMs such as mycotoxins in fungal endophytes. *Alternaria* sp., an endophytic fungus isolated from *Datura stramonium*, when grown in the presence of 250 µM of 5-azacytidine and/or 500 µM of SAHA, produced five compounds that were not detected in the control culture [126]. These compounds included a mycotoxin alternariol (**48**) and two other derivatives, namely, alternariol-5-O-methyl ether (**49**) and 3′-hydroxy-5-methoxyalternariol (**50**), in addition to two other compounds, altenusin (**51**) and Compound (**52**), designated as (5S, 8S)-tenuazonic acid, an *Alternaria* toxin [126]. The growth of the fungus in the presence of the epigenetic modifiers additionally resulted in increased accumulation of a second *Alternaria* toxin, altertoxin II (**53**). In another study, growth of *Dimorphosporicola tragani*, an endophytic fungus isolated from *Arthrocnemum macrostachyum*, in the presence of 5-azacytidine and valproic acid, resulted in the induced biosynthesis of a numbers of compounds. Among these were three toxic compounds, dendrodolide E (**54**), G (**55**]) and I (**56**), that were not produced by the fungus grown in the absence of the two chemical elicitors [127]. 

### 6.4. Molecular-Based Approaches 

The strategies for the activation of the silent BGCs described so far are, generally, untargeted in nature and, thus, do not explicitly permit an association between a newly identified secondary metabolite with any particular gene cluster. Instead, these approaches are meant to activate putative biosynthetic pathways whose identity or number may not be known beforehand. Genetic engineering of target strains is a more precise avenue that can be exploited to awaken cryptic BGCs, an approach that relies largely on genome mining. This approach is BGC specific and first involves the ‘mining’ of the whole genome of the target strains for the typical secondary metabolite gene clusters using appropriate bioinformatics tools [128,129]. Based on the architecture of the identified biosynthetic pathways, appropriate genetic manipulations of the strain of interest are carried out using any of the appropriate synthetic biology tools. Regulation of fungal SMs biosynthesis is a complex process that consists of interconnected pathways mediated by cluster-specific and global transcriptional complexes [83,130]. Pathway-specific regulatory genes which may be located within or outside a specific BGC are involved in the inactivation or repression of biosynthesis of SMs associated with a particular BGC [131]. Beside the pathway-specific regulators, the stimulation of global transcription factors is another possible approach. Global transcription factors which regulate genes that are not involved in secondary metabolism are coded for by genes outside the cluster region and regulate fungal response to various environmental signals such as nutrients, light, pH and stress [99,132]. 

Manipulation of pathway-specific regulators is an especially promising approach considering that up to 50% of fungal BCG have been found to harbor cluster-specific transcription factors [83]. The overexpression of such regulatory factors can result in the expression of cryptic pathways leading to the production of new metabolites. This approach was employed in *Aspergillus nidulans* through the overexpression of transcription factor gene *apdR*, resulting in the biosynthesis of two novel PKS-NRPS hybrid metabolites designated as aspyridone A and B that had not been previously isolated from this fungus [133]. Using the same approach, the activation of *azaR* in *A. niger* resulted in the production of six new azaphilone compounds designated as azanigerones A-F [134]. Similarly, the deletion of transcriptional repressors has also been used to enhance the biosynthesis of SMs or promote the production new fungal secondary metabolites. For example, deletion of *xpp1*, while resulting in the downregulation of primary metabolism, was found to enhance the upregulation of secondary metabolism, leading to significant increases in the quantity and diversity of SMs in *Trichoderma reesei* [135]. In another study, a double deletion of *PfCclA* and *PfcclA*, two epigenetic-related genes in endophytic fungus *Pestalotiopsis fici*, led to the biosynthesis of fifteen novel polyketides, pestaloficiols T-W, 11 macrodiolide ficiolides A-K and ficipyrone C [136]. In addition to the overexpression BCG-specific transcriptional factors and deletion of repressors, the exchange of native promoters with inducible or constitutive promoters is another strategy that has been employed to awaken cryptic BGC in fungi [137]. This approach was notably employed for the development of a system for heterologous expression of BGCs under the control of regulatable promoters and validated through the expression of numerous *Aspergillus terreus* BGCs in *A. nidulans* [138]. The identification and manipulation of negative regulators of SM clusters is another approach used to enhance the secondary metabolite biosynthesis in fungi. Multicluster regulator A (*McrA*) was uncovered as a gene involved in global downregulation of secondary metabolism in *A. nidulans* [139]. The deletion of *McrA* from *A. nidulans* and its homologs in a number of other fungal strains led to the upregulated production of several secondary metabolites, while its overexpression led to the suppression of SM biosynthesis [139]. The overexpression of global positive regulators has likewise been used to enhance SM production in fungi. For example, the overexpression of *LaeA*, a positive regulator found in *Aspergillus* spp., has been shown to enhance SM biosynthesis in members of this genus [140]. Other molecular-based strategies for the activation of silent BGCs in fungal endophytes have been extensively reviewed elsewhere and include those categorized as pleiotropic approaches, such as manipulation of global regulators and ribosome [1], and heterologous host transfer [99,138]. 

It is worth mentioning that the choice of any of the approaches discussed herein will depend upon the requirements of individual researchers, the level of expertise and the nature of the fungal strains, among other factors. None of the approaches can be considered as superior, as each has its strengths and limitations.

## 7. Conclusions and Future Prospects

Endophytic fungi have emerged as promising resources with enormous potential in drug discovery. With only a small fraction of the more than 1 million estimated fungal endophytes investigated for their biosynthetic capacity, fungal endophytes remain a largely understudied resource for the discovery novel bioactive molecules. The discovery of endophytic fungal strains capable of producing plant-associated molecules raises the prospects of exploiting such strains as an alternative source of valuable compounds. For example, the discovery of paclitaxel-producing fungal endophytes paved the way for the production of paclitaxel through a semisynthetic process that involves 10-deacetyl-baccatin III as a precursor, plant cell culture and the endophytic fungi. Such an approach for co-cultivation of fungal endophytes with plant cells may offer the possibilities for production of other useful bioactive compounds that are produced in unsustainable quantities in plants.

However, to derive the immense benefits and possibilities that fungal endophytes promise, a number of constraints need to be addressed. Among the limitations associated with bioprospecting for bioactives from fungal endophytes is the issue of unculturable fungal strains. While the diversification of isolation media has been shown to enhance the recovery rate of cultivable fungal endophytes, numerous studies have revealed that many endophytic fungal strains are never recovered from plant materials because they are unculturable. Although metagenomics techniques facilitate the detection of such strains, it remains a big challenge to assess such strains for their full SMs biosynthetic competencies using the available technologies. Moreover, even among the culturable strains, loss of viability or capacity for biosynthesis of the compounds of interest either as a result of long-term preservation and repeated sub-culturing is a common challenge. This has often led to the loss of promising fungal strains before they are well studied, and attempts at re-isolation of the same fungal strains from the original habitats are not always met with success. Lastly, the future of fungal endophytes as a source of bioactives is tied to that of their host plants. There is a positive correlation between plant diversity and endophytic fungal diversity. The increase in habitat loss and overexploitation of plants resources in different parts of the world are putting many plant species at a risk of extinction. The loss of such plants means a corresponding loss of an assortment of valuable fungal endophytes, many of which may be uncharacterized and potential producers of interesting chemical molecules.

## Figures and Tables

**Table 1:** Some fungal endophytes that have been reported to produce similar bioactive compounds as their host plants.

Endophytic Fungi	Host Plant	Bioactive Compound	Structure	Bioactivity	Reference
*Altenaria alternata*	*Passiflora incarnata*	Chrysin	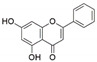	Antibacterial, anti-inflammatory, anticancer effects	[43]
*Alternaria alternata*, *Phomopsis* sp. and *Fomitopsis* sp.	*Miquelia dentata*	Camptothecine	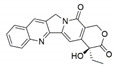	Anticancer agent	[44]
*Aspergillus flavus*	*Solanum nigrum*	Solamargine	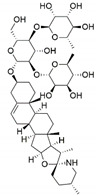	Anticancer activity	[45]
*Aspergillus nidulans*, and *Aspergillus oryzae*	*Ginkgo biloba*	Quercetin	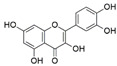	Anti-inflammatory	[46]
*Chaetomium globosum*	*Hypericum perforatum*	Hypericin	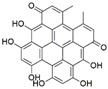	Anti-inflammatory effects, antimicrobialand antioxidant activities	[47]
*Chaetomium globosum*	*Hypericum perforatum*	Emodin	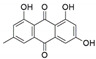	Anti-inflammatory effects, antimicrobialand antioxidant activities,	[47]
*Chaetomium globosum*	*Ginkgo biloba*	Quercetin	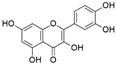	Anti-inflammatory and antiallergic effects	[48]
*Colletotrichum gloeosporioides*	*Forsythia suspensa*	Phillyrin	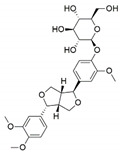	Antioxidant, anti-infl ammatory, anti-hyperlipidemia and antipyretic activities	[49]
*Colletotrichum gloeosporioides* and *Periconia* sp.	*Piper longum* and *Piper nigrum*	Piperine	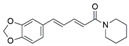	Antibacterial, antifungal, anti-inflammatory and and antioxidant	[50,51]
*Epicoccum nigrum*	*Hypericum perforatum*	Emodin	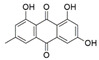	Antimicrobial, anti-inflammatory and antioxidant	[52]
*Fusarium* sp. and *Paecilomyces tenuis*	*Huperzia serrata*	Huperzine A	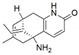	Treatment of Alzheimer’s disease	[53,54]
*Fusarium* sp.	*Mentha longifolia*	Fusaripeptide A	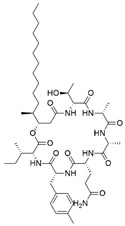	Antifungal, antimalarial and cytotoxicity	[55]
*Fusarium oxysporum*	*Sabina recurva*	Podophyllotoxin	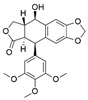	Anticancer agent	[56]
*Fusarium oxysporum*	*Catharanthus roseus*	Vinblastine	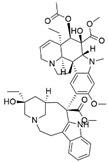	Anticancer/antitumor agent	[57]
*Fusarium oxysporum*	*Catharanthus roseus*	Vincristine	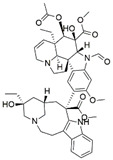	Anticancer/antitumor agent	[57]
*Fusarium proliferatum*	*Macleaya cordata*	Sanguinarine	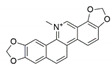	antibacterial, antihelmintic, antitumor	[58]
*Fusarium solani*	*Camptotheca acuminate* and *Apodytes dimidiata*	Camptothecine	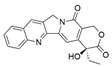	Antineoplastic	[44,59]
*Fusarium solani, Metarhizium anisopliae* and *Mucor rouxianus*	*Taxus chinensis*	Paclitaxel	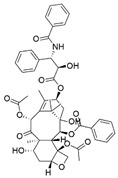	Anticancer/antitumor agent	[60]
*Hypocrea lixii*	*Cajanus cajan*	Cajanol	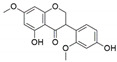	Antiplasmodial, antimicrobial, anticancer agent	[61]
*Pestalotiopsis pauciseta*	*Cardiospermum helicacabum*	Paclitaxel	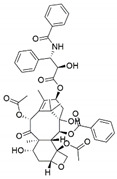	Anticancer/antitumor agent	[62]
*Pestalotiopsis terminaliae*	*Terminalia arjuna*	Paclitaxel	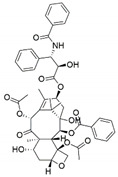	Anticancer/antitumor agent	[60]
*Phialocephala fortinii*	*Podophyllum peltatum*	Podophyllotoxin	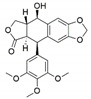	Anticancer, antiviral, antioxidant, antibacterial and anti-rheumatic activities	[63]
*Phoma glomerata*	*Salvia miltiorrhiza*	Salvianolic acid C	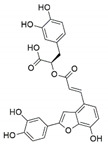	Cardiovascular/ cerebrovascular	[64]
*Phomopsis* sp. and *Diaporthe* sp.	*Cinchona ledgeriana*	Quinine	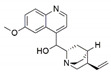	Antipyretic and antimalarial	[65]
*Phomopsis* sp. and *Diaporthe* sp.	*Cinchona ledgeriana*	Quinidine	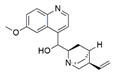	Antipyretic and antimalarial	[65]
*Phomopsis* sp. and *Diaporthe* sp.	*Cinchona ledgeriana*	Cinchonidine	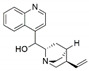	Antipyretic and antimalarial	[65]
*Phomopsis* sp. and *Diaporthe* sp.	*Cinchona ledgeriana*	Cinchonine	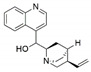	Antipyretic and antimalarial	[65]
*Sordariomycetes* sp.	*Eucommia ulmoides*	Chlorogenic acid	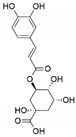	Antimicrobial and antitumor	[66]
*Trichoderma atroviride*	*Salvia miltiorrhiza*	Tanshinone I	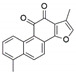	Antibacterial and anti-inflammatory	[67]
*Trichoderma atroviride*	*Salvia miltiorrhiza*	Tanshinone IIA	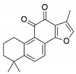	Antibacterial and anti-inflammatory	[67]
*Thielavia subthermophila*	*Hypericum perforatum*	Emodin	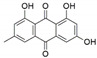	Antimicrobial, anti-inflammatory and antioxidant	[59]

## Data Availability

Not applicable.
